# Experiences and perspectives of UK speech and language therapists on telehealth assessment with people living with post‐stroke aphasia

**DOI:** 10.1111/1460-6984.70018

**Published:** 2025-02-23

**Authors:** Amanda Comer, Sarah Northcott, Nicholas Behn, Abi Roper, Niamh Devane, Katerina Hilari

**Affiliations:** ^1^ Centre for Language and Communication Science Research, School of Health and Psychological Sciences City St George's University of London London UK

**Keywords:** aphasia, assessment, telehealth, qualitative research, speech–language therapy

## Abstract

**Background:**

Stroke care in the UK was significantly affected by the COVID‐19 pandemic, with many services switching to telehealth. Post‐pandemic, a UK survey of speech and language therapists (SLTs) working with people with aphasia (PWA) showed the vast majority planned to continue to use telehealth alongside in‐person intervention. Telehealth is considered a cost‐effective and feasible method of service delivery; however, there is limited evidence to support its use in the assessment of people with post‐stroke aphasia.

**Aims:**

To investigate what barriers and facilitators SLTs experience when administering telehealth assessments to PWA and to explore SLTs’ perspectives on what makes for a positive patient experience.

**Methods & Procedures:**

Focus groups (dyadic/triadic) were conducted via videoconferencing. Transcripts were analysed using framework analysis. Inclusion criteria for participants were SLTs working in the UK with PWA, with experience of using telehealth assessment.

**Outcomes & Results:**

A total of 14 SLTs participated across six groups. Seven themes were identified: assessment; technology; factors specific to PWA; factors specific to family, carers and their environment; factors specific to SLTs; benefits of telehealth assessment; and what telehealth would look like in an ideal world. Facilitators to telehealth assessment included good internet connectivity, access to a helper, adapted assessments, preparation and training PWA to use telehealth platforms. Barriers included reduced control over the environment, having a cognitive impairment, aphasia severity, low beliefs in competence using technology and challenges with managing the emotional needs of PWA during telehealth assessment. A strong therapeutic relationship, offering choice and flexibility in assessment administration, promoted a positive patient experience.

**Conclusions & Implications:**

This study provides new insights into the current use of telehealth assessment with PWA by SLTs in the UK. Barriers and facilitators identified can support the implementation of telehealth assessment in SLT services. Providing a positive patient experience when using telehealth assessment is important to SLTs, with patient choice a key factor. Further research is indicated to increase the range of standardized assessments for telehealth assessment and investigate the efficacy of a hybrid model approach to service delivery.

**WHAT THIS PAPER ADDS:**

**What is already known on the subject**

- There is emerging evidence for the use of telehealth assessment as a feasible and appropriate means of service delivery for SLTs. However, little is known about SLTs’ experience of delivering telehealth assessments to PWA.

**What this paper adds to existing knowledge**

- This study identified both the facilitators and barriers experienced by SLTs when using telehealth assessment with PWA. Practical advice and considerations for telehealth administration are provided.

**What are the potential or actual clinical implications of this work?**

- Resources are required for the implementation of SLT telehealth assessments, particularly in training for both PWA and SLTs, assessments designed for telehealth and accessible telehealth platforms. A hybrid approach to assessment could be beneficial for both PWA and clinicians, retaining the advantages with the acknowledgement that telehealth may not be suitable for all.

## INTRODUCTION

Aphasia is a language disability that can affect understanding, speaking, reading, and writing, and impacts approximately one‐third of stroke survivors (Flowers et al., 2016). During the COVID‐19 global pandemic, usual healthcare delivery was altered. Stroke care in the UK was impacted by the pandemic with fewer stroke hospital admissions (Douiri et al., 2021), and community rehabilitation pathways reduced or stopped in some areas of the UK (Ford et al., 2020). In a survey by the Royal College of Speech and Language Therapists (RCSLT) during the first COVID‐19 lockdown (March–June 2020), 81% of adults with a communication disability reported they received less speech and language therapy (SLT) and 62% did not receive any intervention, with a reduction in neurorehabilitation patients accessing SLT services (Clegg et al., 2021). Evidence suggests that since the end of the initial UK COVID‐19 lockdown, over half of people with aphasia (PWA) reported a decline in their mental health, social life, friendships and home life (Clegg et al., 2021). The challenges of living with aphasia are well described, with PWA more likely to experience poor quality of life and reduced levels of activity (Hilari, 2011). Considering the reported decline in psychosocial functioning and disrupted or lack of service provision, the impact on PWA is likely to be substantial. The COVID‐19 pandemic accelerated implementation of telehealth within the UK and beyond to provide access to healthcare services, including stroke care, whilst reducing face‐to‐face consultations (World Health Organization (WHO), 2021).

Telehealth is the remote provision of healthcare services via telecommunications technology (Oxford English Dictionary, 2022). Telehealth in this paper is defined as synchronous videoconferencing where both clinician and recipient interact with one another in real time.

Telehealth is suggested as an acceptable and feasible way of delivering SLT services to PWA which complement the pre‐existing care model (Cetinkaya et al, 2024). Benefits of telehealth include improved access to care in rural areas, reduced travel burden and enabled patient‐centred care (i.e., increased family involvement, and care in own home; Molini‐Avejonas et al., 2015). Equitable service to face‐to‐face appointments, reduced service provider and user costs, and reduced waiting times are also reported advantages (Weidner & Lowman, 2020). National SLT organisations have responded by developing guidance to promote safe telehealth service delivery, including the RCSLT (2022).

Barriers to healthcare services to implementation of telehealth include variable digital literacy, availability of technology for those considered vulnerable or with low socio‐economic status, sustainable funding, and service and user preference (WHO, 2021). Those with post‐stroke aphasia are likely to experience these barriers, with stroke burden higher in those living in low socio‐economic status areas (Avan et al., 2019).

The RCSLT found early adoption of telehealth using videoconferencing (43.6%) was one of the most common changes made during the COVID‐19 pandemic (RCSLT, 2020). More than half of SLTs reported this pandemic provided opportunities for innovative practice (61%), and 54.4% found they had developed new skills. In addition, Hilari et al. (2023) found 50% of SLTs wanted to see more PWA online, highlighting the flexibility and creativity of SLT service delivery. Telehealth remains a priority for the National Health Service (NHS) as part of its current Long‐Term Plan; however, there remains inconsistent implementation across England and the UK (NHS England, 2019).

Assessing PWA is important for diagnosis, goal setting, directing treatment, providing feedback and measuring outcomes (Bruce & Edmundson, 2010). In recent years, research investigating telehealth assessment has emerged, comparing existing face‐to‐face assessments with telehealth administration. One example is the Western Aphasia Battery—Revised, a recommended impairment‐based outcome measure for PWA (Wallace et al., 2019), where no significant differences were found between face‐to‐face and telehealth delivery (Dekhtyar et al., 2020). A systematic review by Hall et al. (2013) compared telehealth assessment with face‐to‐face assessment, with four studies including PWA. The authors reported equivalence between face‐to‐face and telehealth assessment administration, except when using the Boston Naming Test due to challenges with scoring (Hill et al., 2009). There remains limited evidence regarding the use of telehealth assessment and how to facilitate this for PWA, particularly when addressing their psychosocial needs (Hall et al., 2013).

While PWA report high satisfaction with impairment‐based telehealth assessment (Molini‐Avejonas et al., 2015), challenges remain. Potential challenges to the delivery of telehealth assessment include lack of standard procedures, availability of high bandwidth internet connections, dependence on the skill of the facilitator and service user, and accessibility and availability of technology (Cetinkaya et al., 2024; White et al., 2022). It is suggested that institutional or cultural barriers to the use of technology exist and are not easily overcome by the provision of devices alone (WHO, 2021).

Little research has investigated the SLTs’ experience of delivering telehealth assessments, and their views on what strategies and support are most effective for people living with post‐stroke aphasia. Given the increasing need to deliver cost‐effective, timely assessments to PWA in post‐COVID‐19 healthcare systems, it is important to understand this.

The specific aims of this study are to investigate the following:

- What barriers and facilitators do SLTs experience when administering telehealth assessments to PWA?
- What are SLTs’ perspectives on what makes a positive patient experience in telehealth assessment?

## METHODS

### Design

A qualitative thematic analysis approach with a focus group methodology was used to gather SLTs’ opinions on their experiences of telehealth assessment. This approach allows for the gathering of diverse, rich data and the opportunity to discover new perspectives on this process through participant interactions (Finch et al., 2014). The Standards for Reporting Qualitative Research checklist was used to ensure transparency in reporting (O'Brien et al., 2014; see Supplementary Material 1 for the completed checklist).

### Ethics

Ethical approval for this study was given by City, St George’s University of London School of Health Sciences Research Committee (ethics reference: ETH2122‐1499). All individuals who participated in this study gave informed voluntary consent. Names and identifying details have been changed to maintain anonymity.

### Participant recruitment

Participants were recruited through advertisements on Twitter, the research team's professional networks and SLT clinical excellence networks (i.e., RCSLT affiliated Computers in Therapy clinical excellence network, colleagues known to work with PWA). Those interested were invited to contact the first author to participate, who screened against the inclusion criteria via email or phone call. Those interested were sent the participant information sheet via email. Participants had at least a week to consider their involvement in the study with consent forms signed electronically if they wanted to proceed. Convenience, purposive and snowball sampling were used to optimise recruitment potential (Clark et al., 2021). Data sufficiency was indicated by between four and eight groups, as they were homogenous with a focused topic (Hennink & Kaiser, 2022). The study aimed to achieve a sample size of 24 participants, intending to reach a recommended group size of six participants (Finch et al., 2014). Inclusion criteria were: Health & Care Professions Council (HCPC)‐registered SLTs, working in the UK, working with PWA for all or part of their role, having experience using telehealth assessment with PWA post‐stroke, and being an English speaker with sufficient language skills to participate in the focus group. Exclusion criteria were: SLTs with no experience in delivering telehealth assessments to PWA, those exceeding the current UK retirement age of 66 years old, and student SLTs. Participants were asked to respect the confidentiality of other participants’ identities, views and experiences within the group.

Participants were provided with written study information and offered an informal meeting to discuss their involvement in the study. Once informed consent was gained, participants completed a brief quantitative questionnaire to gather demographic data and information about their SLT role.

### Data collection

Focus groups were co‐facilitated by the first and a senior researcher (second author, a qualitative researcher with experience of conducting focus groups). Both facilitators were female and HCPC‐registered practicing SLTs, with 9 and 21 years of experience working with PWA, respectively. Both facilitators had experience of telehealth and face‐to‐face assessment with PWA. Facilitators knew some participants prior to recruitment, which is an acknowledged source of bias. To promote neutrality and transparency, acknowledgement of these relationships and how they may impact on facilitation of the focus groups was reflected upon prior to commencing, and after each focus group (Ormston et al., 2014). This also applied to the clinical experiences and opinions of the group facilitators. Credibility of findings was supported by the first author keeping a reflexive diary, and through discussion with the research team during data collection and analysis.

Groups were held online via the videoconferencing platform Zoom, with facilitators in private and quiet environments for each group. Each focus group was on average 53.38 min and was audio and video‐recorded using Zoom. Field notes were taken by both facilitators and discussed during post‐group debrief sessions. Dyadic and triadic groups were carried out to facilitate participant involvement in the study in response to slow participant recruitment, by holding groups at convenient times and flexibility for rescheduling due to clinical or personal commitments.

The focus groups were semi‐structured, asking open‐ended questions with additional probing to encourage further reflection. The facilitators encouraged a full range of views and opinions, including contrasting opinions, from all participants with reassurance provided at the beginning of each group that there are no right or wrong answers. A topic guide was used to ensure data gathered focused on the aims of the study (see Appendix A). The topic guide was informed by the results of a recent survey on telehealth (Hilari et al., 2023) and developed through an iterative process of consultation within the research team. The guide was amended after the first focus group was conducted.

### Data analysis

Information on participant characteristics was gathered using a Qualtrics XM online survey, collected after each group. All focus group recordings were transcribed verbatim using transcription software and then reviewed for accuracy by the first author. Anonymity was protected during this process by assigning codes to participants known only to the first author.

Data were analysed using framework analysis (Ritchie & Spencer, 1994). This method is used in qualitative health research and has a clear structure and systematic approach to promote transparency of thematic development (Gale et al., 2013). An inductive approach was used to derive themes from the data (Ormston et al., 2014). The first author initially familiarised herself with data by reading transcripts multiple times and identifying recurring or substantive topics. A thematic index was then developed by the first author, and further refined through discussion with the research team (see Appendix B for the thematic index). Data were indexed by assigning codes to each transcript. This was carried out by the first author and reviewed by the second author after two focus groups were coded to explore theme development. Themes from the analytical process were reviewed and discussed with the wider research team. Coded data were synthesized and summarized into thematic matrices, with each main theme a separate matrix. A participant‐based group analysis approach was used to analyse the phenomena, with individual's data assigned its own row in charting (Spencer et al, 2014). Finally, interpretation of the data was carried out with the aim of mapping the connections within the data, exploring rationales and implications of the phenomena. The first author completed the charting and thematic interpretation, with review from and discussion with the research team (Spencer et al., 2014). Data were managed using Microsoft Excel. This analytic method enabled triangulation of the data through multiple person analysis, with investigator triangulation used within the research team to minimize bias (Denzin, 2009).

## RESULTS

### Participant characteristics

A total of 14 SLTs participated in six focus groups (two groups with three participants, and four groups with two participants) between August and December 2022. Table 1 describes the participant characteristics. All participants identified as female and most reported their ethnicity as White (93%), with 7% as Mixed. Half of the participants were working in community services, with 43% working for a private company or self‐employed and 57% working for the NHS. Most participants (86%) reported working in London and the South East of England, with participants also located in the North West of England and Wales.

**TABLE 1:** Participant demographics (*N* = 14).

**Variables**	***N***	**%**
*Age*		
20–30 years	3	21.43%
31–40 years	5	35.71%
41–50 years	3	21.43%
51–60 years	3	21.43%
61+ years	0	0%
*Gender*		
Male	0	0%
Female	14	100%
Non‐binary/third gender	0	0%
Other and prefer not to say	0	0%
*Ethnicity*		
Asian	0	0%
Black	0	0%
White	13	92.86%
Mixed	1	7.14%
Other	0	0%
*Years of experience working with PWA*		
< 5 years	3	21.43%
6–10 years	3	21.43%
11–15 years	4	28.57%
16–20 years	1	7.14%
20+ years	3	21.43%
*Employer*		
National Health Service (NHS)	8	57.14%
Private company	1	7.14%
Self‐employed	5	35.71%
		
*Work setting*		
Acute hospital	0	0%
Inpatient rehabilitation	2	9.09%
Community/domiciliary	11	50%
Early supported discharge	3	13.64%
Outpatient	3	13.64%
Other^a^	3	13.64%
*% of the role working with PWA*		
0–25%	2	14.29%
2%–50%	4	28.57%
50–75%	8	57.14%
75–100%	0	0%

*Note*: ^a^Specified by participants as: university; completely remote working; and private practice.

PWA, people with aphasia.

### Main findings

**FIGURE 1:**
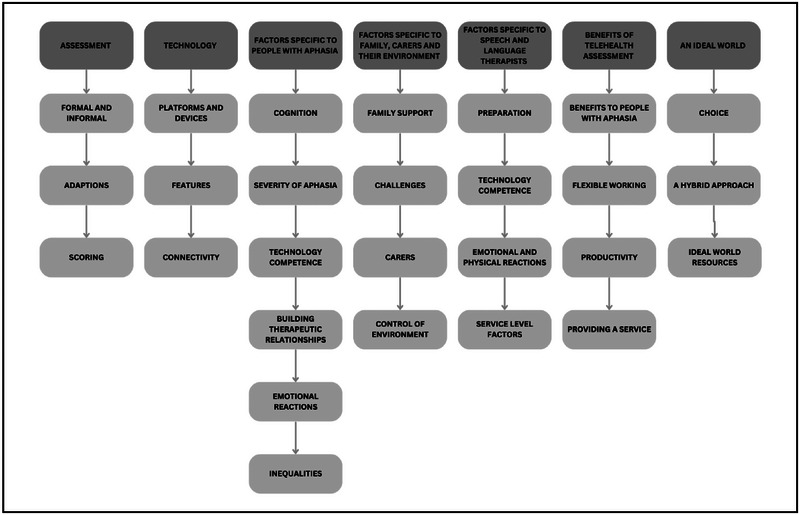
Thematic map.

Seven main themes captured the experiences and factors encountered by participants using telehealth assessment. The themes identified were: assessment; technology; factors specific to PWA; factors specific to family, carers and their environment; factors specific to SLTs; benefits of telehealth assessment; and an ideal world (see Figure 1 for themes and sub‐themes). See Appendix C for a summary of barriers and facilitators identified within themes.

### Assessment

Perspectives differed as to whether telehealth assessment lends itself more to formal or informal assessment. The structure of formal assessment was reported to suit telehealth platforms, however informal functional assessments that were quick to set up and easier for some PWA to access were preferred by participants (e.g., verbal description of the environment). Assessments dependent on spoken output were reported to be less challenging to present as they require fewer resources and adaptations (e.g., fluency measures). Visual assessment items were identified as simple to screen share (e.g., stimuli for spoken picture description), however, viewing items in the person's environment could be challenging. Difficulties changing the camera position to observe pointing or items for single‐word comprehension tasks were identified: ‘You could use the visuals as you're sharing your screen … there was more difficulty when it was too receptive elements’ (Kirsty).

Adapting the presentation of assessments and how PWA could respond was described, for example, rating scales were reported to be easy to adapt and mark responses. Concerns regarding the legality of scanning and adapting assessments, and the reliability of PWA's responses were reported: ‘You're reliant on someone else being there or them all agreeing on a number system and them showing you which one. But it all feels a bit more unreliable if that's what you are using’ (Kath).

Test materials were sent either in advance or shown at the time of assessment, depending on the task need and PWA's competency. The scoring of assessments was described as similar to face‐to‐face delivery. Some participants reported telehealth assessment was easier as PWA were not distracted by the SLT writing responses during the assessment and found ‘the documentation slightly more ordered and structured as well’ (Natalie).

### Technology

A total of 13 participants reported the UK COVID‐19 lockdown prompted their initial use of telehealth assessment, except one participant who reported ‘a few online sessions’ (Liz) prior to the pandemic. Post‐pandemic, most participants described using telehealth assessment less often. The delivery of telehealth assessment using a laptop was preferred by SLTs, with a simple one‐click access to the videoconferencing platform: ‘We would always recommend iPads for being really intuitive to use, but actually Zoom on iPad is really hard, so hard and it's so much, so much easier to join on a laptop for Zoom’ (Liz).

Challenges of videoconferencing platforms used for telehealth assessment included the inability to control the device at the PWA's end, to resolve technical difficulties or to view PWA responses. Setting up the platform for telehealth assessment was reported to be time‐consuming and difficult for PWA. Camera positioning was also perceived by most groups as a challenge. See Table 2 for platforms used by participants.

**TABLE 2:** Videoconferencing platforms used by participants for telehealth.

**Platform used for telehealth assessment**	**Number of participants used/using the platform**
Zoom	11
Microsoft Teams	10
Attend Anywhere	6
WhatsApp	5
Skype	4
Accurx	2
Bluejeans	2
Google Meets	1

Platform features that facilitated telehealth assessment included screen sharing, which was commonly identified as important. Screen mirroring (screen sharing an iPad or iPhone not in the video meeting) was described by a subset as useful for informal assessment using therapy apps and to view multimodality communication strategies. Group calls enabled family members and interpreters to remotely join assessments. The use of recording and screenshots was reported as an efficient way to gather outcome measures (e.g., PWA typing on the chat function or recording discourse samples). Annotate was another frequently mentioned tool to facilitate telehealth assessment:

When you tap that one um a little dot comes up on it so I can see which one they tap, or they click on it, and so are you like they can scribble on it … I find that really helpful for a formal assessment point of view. (Susie)

### Factors specific to PWA

Cognition was perceived as a significant barrier to telehealth assessment: ‘cognition does impact on someone's ability to engage’ (Jessica). The cognitive load of telehealth assessment, particularly for executive functions such as attention control and problem solving, were reported to be high and required active management by offering shorter assessment sessions, aphasia friendly step‐by‐step guides for telehealth assessment and establishing routines for the set‐up and administration of telehealth assessment. Visual, hearing, and physical impairments were reported to impact on telehealth assessment (e.g., ability to position device/camera).

Severe aphasia was also described as a barrier to telehealth assessment, given telehealth platforms often depend on some verbal communication which may not allow PWA to communicate effectively in their usual way (e.g., drawing or writing). One participant described how aphasia severity was considered in their criteria for telehealth use: ‘People did have to understand at least kind of two key instructions … it's just so frustrating to someone if they've got really severe receptive aphasia to try’ (Mary).

**FIGURE 2:**
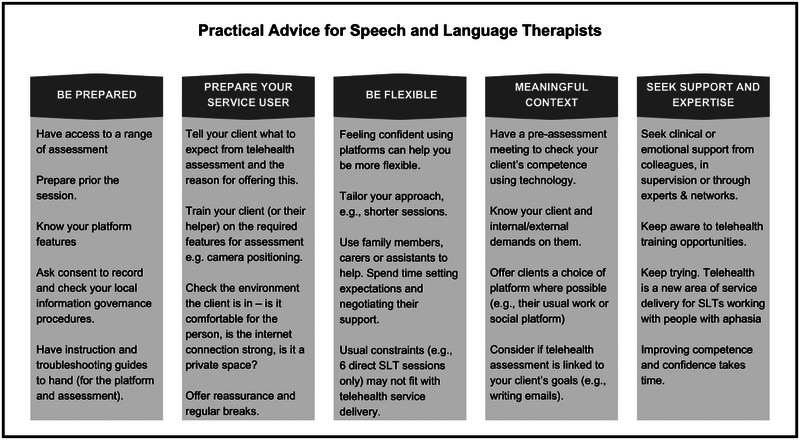
Practical advice derived from data for SLTs carrying out telehealth assessment. Note: SLTs, speech and language therapists.

Competence using technology was perceived as a facilitator when using telehealth assessment. Those living more independently and those younger were thought to have good levels of competence with technology, with older PWA potentially using technology in this way for the first time. Providing opportunities for PWA to frequently use telehealth was perceived to have the potential to improve their confidence and efficacy, however most participants reported PWA having support needs to access telehealth assessment effectively. Understanding the PWA's competence was key to the introduction of telehealth assessment: ‘Knowing what someone's competency first is really important because you don't want to kind of bombard someone with instructions or offend them or patronise them’ (Nicole). See Figure 2 for a summary of practical advice derived from the data.

Most participants reported the need to work harder to build rapport with PWA via telehealth, with delays in connection affecting the dynamic of conversations and ‘interrupted the naturalness of a conversation’ (Sandra). An alternative view was provided by a participant working solely as a remote SLT: ‘You just build up that relationship, as you would um if you were face‐to‐face, no difference really… it's quite sort of natural in that way’ (Margot). A need to put people at ease for telehealth assessment was identified to support participation. Strategies described to build rapport were having an initial meeting face‐to‐face, taking time to talk with PWA before starting telehealth assessment, and working with the same clinician. In addition, the lack of personal protective equipment required was described as a benefit of telehealth, which a subset of participants reported could enhance therapeutic dynamics.

Observing emotional responses and subtle cues were highlighted as more difficult, affecting the support participants felt they could offer:

I think probably is just the same as face to face, but probably a few more barriers in terms of really being able to recognise like the impact of, you know, struggling with assessments or knowing when it's appropriate to kind of adapt your style. Can be a bit harder over the screen. (Charlotte)

Participants reported the introduction of telehealth assessment could cause anxiety for some PWA, with technical difficulties adding to this. PWA's resistance to engage with telehealth and frustration with telehealth assessment was described. Reassurance and regular breaks were suggested by participants as useful strategies to support engagement.

Participants highlighted that inequalities were apparent when telehealth was introduced. Lack of internet, technology and financial barriers affected whether PWA could participate in telehealth assessment. One improvement in equity was access to video interpreting services, with a subset describing this as preferable to telephone interpreting. Another consideration reported was social isolation and the inaccessibility of support networks during the lockdown: ‘What does it tell us about their kind of social isolation as a result of the pandemic if they can't use Zoom’ (Charlotte). A subset of participants identified those with communication needs could be deprioritized and safeguarding risks may be under reported:

There is risks of seeing people just online that you miss the fact that they might be in a challenging environment or they might be safeguarding risks or they, you know, the other things that they disclose to you in those situations that allow you to work in a much more holistic way. (Mary)

### Factors specific to family, carers, and their environment

Family support for PWA was identified as an important facilitator for telehealth assessment: ‘We were relying a lot on, um, family members or people who are present’ (Jessica). The benefits of family support were described as setting up the device and platform, explaining where the PWA were pointing or looking, and facilitating multimodality communication use. Family members were reported being more available for telehealth assessment and could be involved in an observer role: ‘That joining together with the people in your life, was really improved, and by that to be able to share the assessment in real time that I found beneficial’ (Poppy).

Challenges with family support were competence with technology to support PWA, balancing how much time is required, if the family member did not want to help, and if PWA did not want support. Difficulties accessing help at nursing or residential care homes was described by a subset of participants, specifically when different staff members were involved in booking and attending the session.

Gaining control of the assessment environment was identified as important by participants. Strategies included spending time with family members to help them understand their role and asking who was in the room at the beginning of every session. However, lack of privacy, noise and difficulty knowing whether PWA were being prompted by others in the room were reported as challenges. Understanding the impact of these factors on assessment was reported to be difficult by participants:

It's like the extra layer that you just can't control … It's so much harder to do online … Sometimes I find those little. Kind of, um, kind of gentle ways that you might control the environment. Just you can't do over zoom in the same way. (Liz)

### Factors specific to SLTs

The use of telehealth assessment was facilitated by sharing ideas and practical tips within teams, developing instructional and troubleshooting guides, working with students and reaching out to clinicians with expertise (including formal training). Collaborative working with assistants or volunteers to help PWA set up their device for telehealth assessment was also an enabler. Participants reported being drivers of telehealth use within multidisciplinary teams:

My team have been really really helpful and supportive about effectively getting people on. So we will go out of our way to like, go to your house and do a pretend session with you … it's great for the rest of your journey. (Susie)

The barrier to using telehealth by information governance was re‐evaluated by some services at the start of the UK COVID‐19 lockdown, however limitations remained with platform options, lack of individual logins and security restrictions such as locking virtual rooms when PWA entered. Lack of access to devices to carry out telehealth assessment was widely reported as a barrier, with initial delays to technology provision reported. Room availability in clinical settings was identified as a barrier to continued use of telehealth assessment, whereas other participants reported easy access to private rooms. Internet connectivity was also reported as both a facilitator and barrier. One group reported their worksite internet connectivity affected their ability to continue with telehealth assessment. Having a stable internet connection was identified as a key enabler to assessment delivery.

The impact on participants when using telehealth assessment was described, with participants reporting exhaustion, worry for PWA during the pandemic and feeling detached. The inequality of service provision to PWA affected participants, with one stating ‘it's not fair’ (Poppy). Strategies to manage the demands of telehealth included regular breaks, eye rest and opportunities to disconnect throughout the day:

You were used to quite back‐to‐back assessments … say, actually I'm just gonna book every, you know, 15 minutes, half an hour, and just making sure to just to take, take a break when you needed it … so hard to disconnect, um, that first, those first few months in Covid. (Michelle)

The competence of SLTs reportedly affected the delivery of telehealth assessment, with variability in confidence, resilience and skills. Participants reported regular use of telehealth assessment as a facilitator: ‘It's almost become like second nature now’ (Margot). Being recently qualified as an SLT and using telehealth assessment during lockdown was perceived by one participant as challenging. Another participant reported a preference for face‐to‐face assessment describing themselves as a ‘reluctant online therapist’ (Kath).

Allocating time to prepare for telehealth assessment was widely perceived as salient, including having a detailed understanding of the telehealth platform used. Carrying out a pre‐assessment session in person was reported by participants as an important facilitator for telehealth assessment, intending to reduce the impact of technology on performance and an opportunity to provide training to PWA or their helpers: ‘We called it a tech check … So we'd familiarised them with the kind of layout of the sessions, get to know them’ (Nicole). Other facilitators included problem‐solving (e.g., how to approach difficult conversations or how PWA would use physical assessment materials), and minimizing distractions on the clinician device.

### Benefits of telehealth assessment

Flexible working was identified as a benefit for participants. Offering both telehealth and face‐to‐face service delivery, increased access to PWA from greater geographical distances and working at home were described as benefits: ‘That privacy and the space and I had decent enough internet connection and, and like a laptop that I knew well’ (Sandra). Other practical benefits included finding appointment times which were previously unavailable (e.g., early morning appointments as no travel required), no forward planning regarding access to PWA's homes and efficiency of having assessments in one online place.

Increased productivity was reported, primarily due to reduced travel time to PWA's homes: ‘In some ways I think that that can make us more productive and, and more patient‐centered’ (Natalie). The time saved enabled more preparation for sessions, more appointments during the day (including shorter sessions), seeing PWA more regularly and could reduce waiting lists: ‘Do 20 minutes today and 20 minutes tomorrow … it's really much more productive as a team to do it that way’ (Susie). Caseload management approaches included identifying who could be appropriate for telehealth from waiting lists and using telehealth assessment to provide services to communicate caseloads quicker during lockdown.

The time‐saving aspect of telehealth assessment was also reported as a benefit to PWA, with no travel required and reduced fatigue: ‘Potentially seeing the best of them’ (Charlotte). Appointments could be offered at more suitable times, or when PWA were not at home, for example, at work or on holiday. Other perceived benefits were PWA feeling comfortable in their own home, with stressors such as cleaning or travel removed, and the use of a self‐management approach by PWA.

The ability to provide a SLT service during the restrictions of the UK lockdown was widely reported as a benefit. Previous barriers to telehealth assessment were overcome, partly through resource provision, and enabled access to those who were clinically vulnerable. The ability to continue to offer student placements was also identified as a positive outcome.

### An ideal world

Participants identified a variety of resources they perceived would improve telehealth assessment in their practice, including more technology for PWA and SLT services, a space to share information and resources with other clinicians, formal training for the use of telehealth assessment, time to carry out assessment at PWA's pace, and for PWA to have a personalized technology set up session:

I would like to benefit from the experience that people have had in developing these services and what works and what doesn't work … rather than just kind of using it as a tool because it's available, making the most of it clinically. (Jessica)

Paper and video guides for clinicians on how to use telehealth platforms and assessments were suggested to promote competence. Access to a wide range of assessments designed for telehealth was highlighted, both formal (including reading assessments and aphasia assessments in other languages) and informal assessments. A subset of participants proposed a method of telehealth assessment delivery via a platform where assessments are stored and can be accessed by both clinicians and PWA, observing PWA responses in real time or reviewed later: ‘Having online or a platform that could produce the assessment stimuli and pictures … where the patient goes on to the application and completes the assessment’ (Sandra).

The importance of offering choice and seeing PWA face‐to‐face was identified by all participants. One participant described how they perceived PWA did not value telehealth as highly as face‐to‐face service delivery. Providing PWA with choices was identified as salient, first whether they wanted to use telehealth and which platform they preferred.

Providing a hybrid service was described as beneficial by all participants, for both clinicians and PWA: ‘I think that works really well for a lot of people’ (Charlotte). Descriptions of hybrid approaches varied between participants with some initially using telehealth assessment and others using this once they had met PWA face‐to‐face:

There are lots of bits of different assessments I can do online really, really well. But then I like to then go and do an in‐person assessment to finish up … I can do it in a hybrid way. So I feel like best of both worlds. (Liz)

**FIGURE 3:**
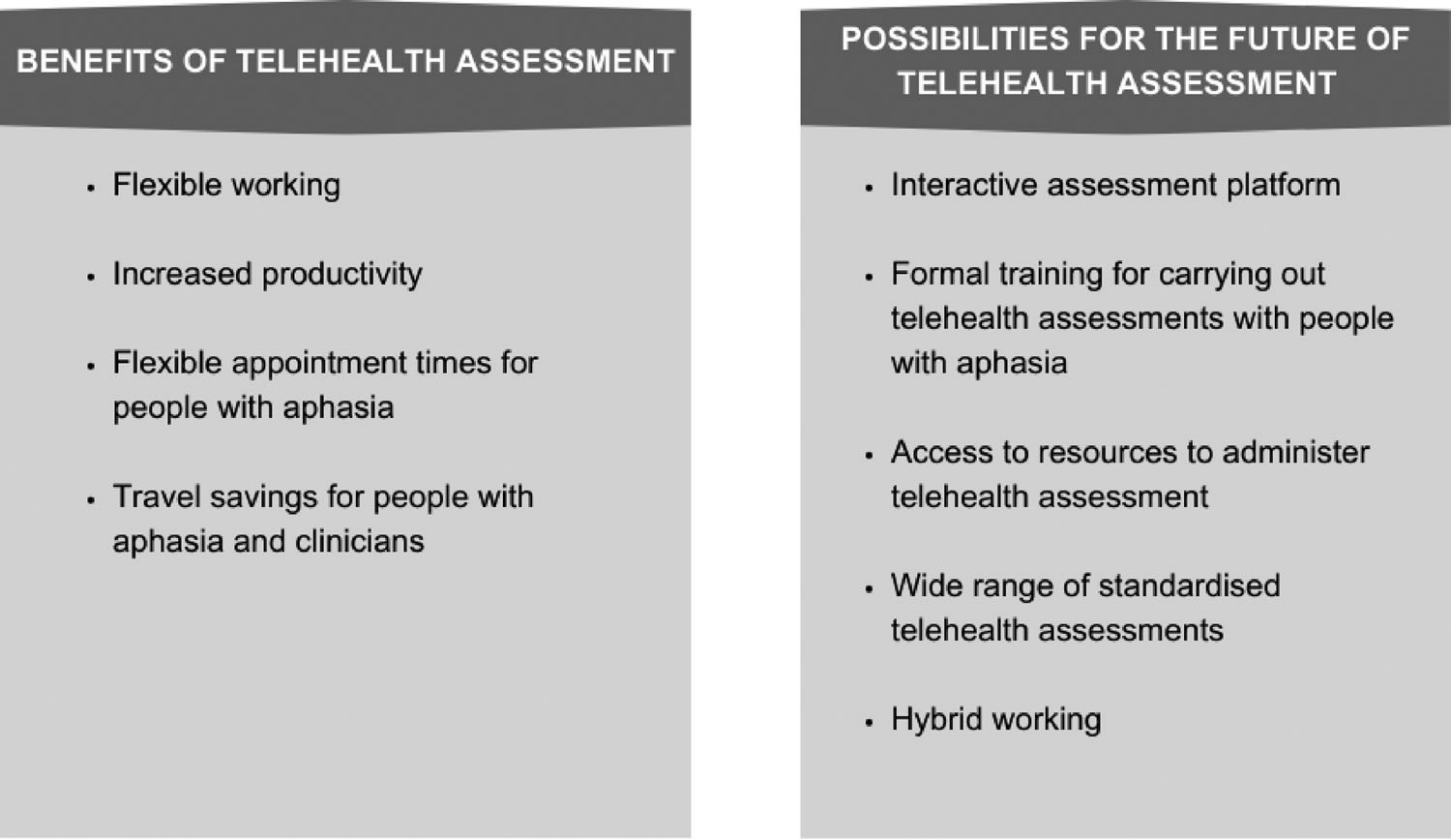
Summary of the benefits and possibilities of telehealth assessment derived from the data.

Telehealth was reported to be included in post‐pandemic current practice in some form, with a subset of participants reporting caseloads as 50:50 between accessing telehealth and face‐to‐face service delivery. Investments in resources for telehealth were identified by a subset of participants: ‘definitely for NHS trusts to recognize that if they want this hybrid model, which is cost‐effective in a lot of ways, they need to invest in resources, systems’ (Charlotte). See Figure 3 for a summary of the benefits of and hopes for the future using telehealth, derived from the data.

## DISCUSSION

This study explores the experiences and perspectives of SLTs using telehealth assessment with PWA. Facilitators to telehealth assessment included access to laptops or tablet devices, a strong internet connection, training PWA to use telehealth platforms, adapted assessments, access to a helper and time to prepare for assessment. Barriers to telehealth assessment were low beliefs in the competence of both PWA and SLTs using technology and telehealth platforms, cognitive impairment, aphasia severity, reduced environment control and managing the emotional demands of assessment. A positive patient experience was reported by delivering a hybrid model of care, offering PWA choice, a strong therapeutic relationship and flexibility of assessment administration.

One of the study's main findings was the importance of preparation for the successful administration of telehealth assessment, for SLTs and PWA. Providing practical training to PWA to upskill them in technology and platform use (e.g., practising key features required for telehealth assessment) could manage the competency effect on assessment and minimise barriers to accessing telehealth (Le et al., 2023), and is highlighted in best practice guidelines (RCSLT, 2022). Preparation for SLTs in setting up their services for telehealth assessment included developing practical resources for PWA and staff, clinical development in telehealth use, and adapting assessments. Resource limitations, such as a stable internet connection and staffing, appeared to impact the scope of preparation carried out by participants. Adapting assessments for telehealth administration appears vital to facilitating telehealth assessment, with efforts made to maintain the presentation of the assessment for normative scoring (Dekhtyar, et al., 2020), yet concerns that these results could be unreliable remained. Verbal naming assessments, for example, the Boston Naming Test, were overall reported as easier to administer as they were deemed simple to adapt, however, evidence suggests that scoring of these tasks was not equivalent to face‐to‐face when delivered via telehealth (Hill et al., 2009). Establishing the latency period and recording sessions to review later may support accurate scoring.

Post‐stroke disability could impact on telehealth assessment administration, with cognitive, physical, sensory and severity of language impairment identified as barriers, however these barriers are not unique to telehealth (Weidner & Lowman, 2020). The use of helpers for PWA during assessment was identified as a significant facilitator to overcoming these barriers. Identifying appropriate family members or carers is important, or rehabilitation assistants and support workers, who could be offered tailored training (Weidner & Lowman, 2020). Similar barriers and facilitators (such as access to resources and clinician confidence) were reported in a study exploring SLTs opinions of telehealth assessment with children (Sutherland et al., 2021). This suggests consistency in factors enabling telehealth assessment across SLT caseloads and services, with the opportunity for parallel growth of telehealth within trusts. Despite NHS recommendations to continue with developments in technology and healthcare made during the COVID‐19 pandemic (NHS England, 2022), there does not appear to have been sustained use of telehealth assessment post pandemic. Most participants working in the NHS described their experience using telehealth during the COVID‐19 pandemic and rarely as current practice. Perceptual and institutional barriers described in the results (i.e., telehealth not feeling the same as in‐person assessments, having severe aphasia and variable resource provision) may have influenced the move away from telehealth service delivery post pandemic. These factors, including forced adoption of service delivery during the pandemic, are described as barriers to continued telehealth use (Thomas et al., 2024). Financial investment in resources for telehealth was identified as a substantive requirement to facilitate use of telehealth assessment. This includes digital inclusion which is a high priority in aphasia research and for national and international healthcare organizations (Ali et al., 2022; WHO, 2021).Incentives for providing telehealth assessment appear driven by the clinician to offer a flexible provision to their service user, which could include improving accessibility of SLT services to PWA, with reduced travel costs and savings to service users (Guo et al., 2014).

The psychosocial aspects of living with aphasia were identified by participants as an important assessment area, which is reflected in recent literature (Cacciante et al, 2021), and is arguably challenging to complete without a therapeutic relationship between PWA and clinicians. Building good therapeutic relationships is suggested to improve a person's experience with healthcare professionals (NHS Institute for Innovation & Improvement, 2013). For PWA, recognizing subtle nuances in conversation, being friendly and open, and seeing the same clinician helps to facilitate strong relationships (Lawton et al., 2018). Participants identified this alliance as a key consideration during telehealth assessment, and how telehealth could impact this (e.g., poor internet connectivity, difficulty recognizing subtle cues through a screen).

Creating a positive service user experience was identified as salient to telehealth assessment. A hybrid model of care delivery, providing a combination of both face‐to‐face and telehealth care, was described as preferable with a focus on person‐centred care and offering meaningful choices to PWA. The use of telehealth with PWA is an evolving area of research, with rehabilitation suggested to be equitable between face‐to‐face and telehealth delivery (Cacciante et al., 2021; Weidner & Lowman, 2020). Building this evidence base, for example, an improved variety of standardized assessments and procedures for the appropriate use of telehealth assessment may support increasing the use of this in SLT services. Developing clinicians’ skills in the use of telehealth assessment with PWA and sharing good‐practice guidance may support with growth of this provision.

### Limitations

Though 14 participants were recruited from a range of settings, we did not reach the planned sample size of 24. Recruitment to focus groups is widely reported as challenging in qualitative health research (Clark et al., 2021). Strategies used to increase the sample size included extending the recruitment period and using videoconferencing to optimize access to SLTs across the UK. The sampling strategy may affect the generalization of the results. Participants were predominantly working in the southeast of England which may not be reflective of the wider UK workforce, and the majority were white and female, which is typical of the UK SLT workforce (HCPC, 2021). The number of participants who were self‐employed or working in the private SLT sector (43%) may not be representative of SLTs working with PWA post‐stroke. Collecting data on specific assessments used by participants and the extent of participants’ experience using telehealth and telehealth assessment could have provided additional context to the results.

Group sizes were small, with dyad and triad composites used, whereas typical focus groups have around six members (Finch et al., 2014). Smaller groups enabled an in‐depth approach whilst maintaining a dynamic of reflection and debate between participants. Other suggested benefits include creating more opportunities for disagreement without the pressure of an outlier opinion which is thought to be more manageable for group moderators (Clark et al., 2021). Two participants had a pre‐existing peer working relationship within the same NHS trust, which may have created a different interaction style compared to the other focus groups.

Respondent validation was not carried out during this study, which is a limitation of the data analysis. The challenges associated with this include new insights which cannot be used in isolation and the potential for participants to request censorship or a reluctance to be critical (Clark et al., 2021).

## CONCLUSIONS

The SLTs in this study identified both barriers and facilitators experienced when using telehealth assessment with PWA. These factors have the potential to inform changes in current practice, with value attributed to the preparation required by PWA, clinicians and services to implement telehealth assessment. A positive patient experience is integral to telehealth assessment success, with SLTs remaining committed to this. A hybrid model to assessment appears to be an important development area in post COVID‐19 healthcare for PWA and clinicians.

## Supporting information



Supplementary Information

## Data Availability

The data that support the findings of this study are available from the corresponding author upon request. The data are not publicly available due to privacy or ethical restrictions.

## APPENDIX A FOCUS GROUP TOPIC GUIDE V.2 (FINAL VERSION)

### Introduction

- Welcome to the group and thank yous
- Researchers’ introduction
- Purpose of focus group—exploring experiences of speech and language therapists using online assessments with people with aphasia, including what a good assessment experience looks like, and the barriers and facilitators to this
- Group guidelines/ground rules—all opinions welcome, no right or wrong answers, confidentiality and data use.
- Recording of group: inform participants the group will be recorded for transcription purposes, ask participants to avoid speaking over each other, start recording.

#### Start recording

- Participant introductions—name, clinical setting, reason/time online assessments were first used, videoconferencing platforms used

#### Facilitators

In your experience, what helps or facilitates online assessments?

- Prompts:

- Technology—connectivity, platform features,
- People with aphasia & family—access to technology, digital literacy and poverty, support to access
- Clinicians—confidence, competence, support to access
- Service—information governance, acceptability, platforms
- Resources available—to SLT/person with aphasia/family member
- Adjustments made by SLT/person with aphasia/family member
- Rapport‐building

#### Barriers

What barriers have you experienced when delivering online assessments?

- Prompts:

- Technology—connectivity, audio/visual difficulties, platform features
- People with aphasia & family—access to technology, digital literacy and poverty, support to access, keeping appointments, severity of aphasia, cognitive impairment, emotional demands
- Clinicians—confidence, competence, support to access
- Service—information governance, acceptability, platforms, DNAs
- Rapport building

#### Experiences & benefits

- What are the benefits of online assessment?
- Ideal conditions for online assessment

- Aspects of assessment process that translate well to online delivery
- Strategies/techniques used
- Resources available

- Online assessments used, inc. psychosocial assessments

#### Closing the group

- Final thoughts

Is there anything we've left out or do you feel you haven't had a chance to say?

What are your top tips for carrying out online assessments?

- Thank yous and closing statements, including confidentiality reminders. Stop recording

**Stop recording**

## APPENDIX B FRAMEWORK METHOD ANALYSIS—THEMATIC INDEX V.3 (FINAL VERSION)

- **Technology**

- Use of OA/videoconferencing and platforms (inc. Initial use)
- Types of devices/equipment used & features of devices/platforms (facilitate or barrier—i.e., touchscreens)
- Wifi/internet connection
- Challenges and barriers with videoconferencing platforms (i.e., limitations in functions)
- Other

- **Assessments**

- OA carried out (i.e., formal/informal, inc. named assessments)
- Scoring and interpreting inc. visibility of PWA response (inc. Total communication)
- Other

- **Factors specific to PWA for OA**

- Access to technology for PWA
- Willingness/engagement for OA (inc. Preparation)
- Competence of PWA with technology
- Techniques or support for PWA (i.e., managing fatigue,1.1 shorter sessions, multiple sessions, environment considerations such as lighting)
- Rapport building and emotional needs during OA
- Use of interpreters
- Physical, cognitive or language (severity of aphasia) requirements, demands or barriers
- Other

- **Family and significant others supporting PWA with OA**

- Access to family/others
- Role within OA sessions (i.e., observer, active supporter)
- Challenges of working with family/other support
- Other

- **Factors specific to SLTs for OA**

- Collaborative working within SLT and wider MDT teams (inc. peer support)
- Competence using technology (i.e., existing, developed)
- Preparation and time for OA (inc. adapting assessments, resource development, setting up environment, doing pre‐ax for OA)
- Negative, emotional and physical impact (inc. cognitive fatigue from screentime, sitting at desk, emotional load)
- Service facilitators & barriers
- Other

- **Benefits of remote working/use of OA**

- Flexibility of working patterns for SLTs
- Caseload management and productivity (i.e., managing workload, improved capacity to see more PWA, reducing waiting lists)
- Convenience, reduced costs and travel for PWA
- Ability to provide service when usual care affected (i.e., lack of physical visits, impact of pandemic, PPE)
- Other

- **‘Ideal world’ for OA**

- PWA choice (inc. flexibility of use of platforms, setting of OA)
- Hybrid model of service delivery
- ‘Ideal world’ resources
- Other

- **Misc/Other**

- Misc/Other thoughts/ideas not addressed above (i.e., background of SLT including setting, online therapy)
- Not relevant
- Other

Key: PWA—person with aphasia; OA—online assessment; SLT—speech and language therapist.
