# Supplementary material for: Experiences and perspectives of UK speech and language therapists on telehealth assessment with people living with post‐stroke aphasia
Source: Int J Lang Commun Disord. 2025 Feb 23;60(2):e70018. doi: 10.1111/1460-6984.70018 (PMC11847568; doi:10.1111/1460-6984.70018)
Supplement: Supplementary file 1 — Supplementary Information [file JLCD-60-0-s001.docx]

**Supplementary material 1**

**Standards for Reporting Qualitative Research (O’Brien et al., 2014) - Checklist for Reader**

| No. | Topic | Item | Location reported |
| --- | --- | --- | --- |
| S1 | **Title** | Concise description of the nature and topic of the study identifying the study as qualitative or indicating the approach (e.g. ethnography, grounded theory) or data collection methods (e.g. interview, focus group) is recommended | Title |
| S2 | **Abstract** | Summary of the key elements of the study using the abstract format of the intended publication; typically includes background, purpose, methods, results and conclusions | Abstract |
|  | **Introduction** |  |  |
| S3 | Problem formulation | Description and significance of the problem / phenomenon studied: review of relevant theory and empirical work; problem statement | Introduction para 1-8 |
| S4 | Purpose or research question | Purpose of the study and specific objectives or questions | Introduction para 7,8 |
|  | **Methods** |  |  |
| S5 | Qualitative approach and research paradigm | Qualitative approach (e.g. ethnography, grounded theory, case study, phenomenology, narrative research) and guiding theory if appropriate; identifying the research paradigm (e.g. postpositivist, constructivist / interpretivist) is also recommended; rationale. The rationale should briefly discuss the justification for choosing that theory, approach, method or technique rather than other options available; the assumptions and limitations implicit in those choices and how those choices influence study conclusions and transferability. As appropriate the rationale for several items might be discussed together. | Method:  Design |
| S6 | Researcher characteristics and reflexivity | Researchers' characteristics that may influence the research, including personal attributes, qualifications / experience, relationship with participants, assumptions and / or presuppositions; potential or actual interaction between researchers' characteristics and the research questions, approach, methods, results and / or transferability | Method  Data collection para 1,2 |
| S7 | Context | Setting / site and salient contextual factors; rationale | Method:  Data collection |
| S8 | Sampling strategy | How and why research participants, documents, or events were selected; criteria for deciding when no further sampling was necessary (e.g. sampling saturation); rationale | Method: Participant recruitment para 1 |
| S9 | Ethical issues pertaining to human subjects | Documentation of approval by an appropriate ethics review board and participant consent, or explanation for lack thereof; other confidentiality and data security issues | Methods: Ethics.  Participant recruitment para 2 |
| S10 | Data collection methods | Types of data collected; details of data collection procedures including (as appropriate) start and stop dates of data collection and analysis, iterative process, triangulation of sources / methods, and modification of procedures in response to evolving study findings; rationale | Method:  Data collection.  Data analysis.  Results: Participants characteristics |
| S11 | Data collection instruments and technology | Description of instruments (e.g. interview guides, questionnaires) and devices (e.g. audio recorders) used for data collection; if / how the instruments(s) changed over the course of the study | Method:  Data collection para 2,3.  Appendix 1 |
| S12 | Units of study | Number and relevant characteristics of participants, documents, or events included in the study; level of participation (could be reported in results) | Results: Participant characteristics, Table 1 |
| S13 | Data processing | Methods for processing data prior to and during analysis, including transcription, data entry, data management and security, verification of data integrity, data coding, and anonymisation / deidentification of excerpts | Methods:  Data analysis |
| S14 | Data analysis | Process by which inferences, themes, etc. were identified and developed, including the researchers involved in data analysis; usually references a specific paradigm or approach; rationale | Methods: Data analysis, para 2. Appendix 2 |
| S15 | Techniques to enhance trustworthiness | Techniques to enhance trustworthiness and credibility of data analysis (e.g. member checking, audit trail, triangulation); rationale | Methods: Data analysis, para 2 |
|  | **Results/findings** |  |  |
| S16 | Synthesis and interpretation | Main findings (e.g. interpretations, inferences, and themes); might include development of a theory or model, or integration with prior research or theory | Results:  Main findings and themes |
| S17 | Links to empirical data | Evidence (e.g. quotes, field notes, text excerpts, photographs) to substantiate analytic findings | Results:  Main findings and themes |
|  | **Discussion** |  |  |
| S18 | Integration with prior work, implications, transferability, and contribution(s) to the field | Short summary of main findings; explanation of how findings and conclusions connect to, support, elaborate on, or challenge conclusions of earlier scholarship; discussion of scope of application / generalizability; identification of unique contributions(s) to scholarship in a discipline or field | Discussion |
| S19 | Limitations | Trustworthiness and limitations of findings | Discussion: Limitations |
|  | **Other** |  |  |
| S20 | Conflicts of interest | Potential sources of influence of perceived influence on study conduct and conclusions; how these were managed | Declaration of interest statement |
| S21 | Funding | Sources of funding and other support; role of funders in data collection, interpretation and reporting | Acknowledgements |
